# Personals

**Published:** 1912-10

**Authors:** 


### PERSONALS.

---

Dr. Edward S. Bogert of the U. S. Navy, has been assigned to temporary duty at the Buffalo station, vice Dr. Andre E. Lee who was transferred to Pekin, China.

---

Major Wm. G. Bissell of the 74th Regiment, Buffalo, has been designated to attend the meeting of the Association of Military Surgeons in Baltimore, Oct. 1.

---

The Board of State Examiners with regard to castration of criminals and defectives consists of Dr. Charles H. Andrews of

Buffalo, surgeon; Dr. Lemon Thompson of Glens Falls, Neurologist; Dr. Charles G. Duryes of Schenectady, general practitioner. An organization meeting was held Sept. 4 and 5 and the board will begin its work promptly by an inspection of the institutions in the eastern part of the state. We refrain from the two obvious puns.

---

Dr. E. W. Mulligan of Rochester, has moved from 318 to 788 East Ave.

---

Dr. Arthur Gillette of New York, was the guest of Dr. H. F. Gillette of Cuba, in September.

---

Dr. M. B. Shaw of Eden, has returned from a trip to Chautauqua.

---

Dr. O. S. McKee of Buffalo, went to Duluth by lake in September.

---

Dr. A. W. Bayliss of Buffalo, sailed for France, Aug. 31.

---

Dr. A. B. Knisley of Buffalo, returned Sept. 1, from a trip to the Thousand Islands and Quebec.

---

Dr. Francesco A. Valanti, who has recently returned from a visit to Europe, has located at 571 Elmwood Ave., Buffalo.

---

Dr. J. A. Rafter, formerly on the editorial staff of the BUFFALO MEDICAL JOURNAL, has been nominated as Democratic candidate for Mayor of North Tonawanda.

---

Dr. Henry Smoyer, a graduate of Niagara University, 1895, has been similarly endorsed for City Treasurer of North Tonawanda.

---

Dr. William Van Russell Blighton of North Tonawanda, has been nominated as Representative in Congress by the Prohibitionists.

---

Dr. L. Bradley Dorr, Councilman in Buffalo, has become a Bull Moose and very honorably declares that he will resign if the forthcoming vote indicates an opposition to the change.

---

Dr. John O. Roe of Rochester, announces his removal to 64 Clinton Ave., South, in association with Dr. Bradford A. Richards. Dr. Roe also sends a very welcome commendation of the growth of this JOURNAL.

Dr. Thomas J. Walsh of Buffalo, will spend the coming fall and winter in study in Germany.

---

Dr. Frederick Frisch who moved from Buffalo to Atlantic City, last year, visited his old home in September.

---

Drs. C. F. Chaffee and Robert T. French of Rochester, have recently sustained fractured wrists from cranking automobiles. Dr. N. D. McDowell of Rochester, sprained his wrist in the same way.

---

Dr. E. H. Wolcott of Rochester has returned from a vacation in the White Mountains.

---

Dr. C. Wentworth Hoyt of Rochester, left for an extended European trip the middle of September.

---

Dr. F. W. Zimmer of Rochester, has recently returned from a trip in the St. Lawrence region.

---

Dr. E. W. Mulligan of Rochester, returned early in September from a camping trip of several weeks in Algonquin Park, Canada.

---

Dr. W. H. Marcy of Buffalo, returned early in September from Webster, Mass.

---

Dr. C. B. Knowlton of Buffalo, camped at Elton, N. Y., during August.

---

Dr. Eli H. Long of Buffalo, announces the removal of his office from 1335 to 1339 Main St.

---

Dr. Herbert Upham Williams of Buffalo, has returned from the White Mountains.

---

Dr. Thomas E. Soules has moved his office from 915 Main St. to 55 Allen St., Buffalo.

---

Dr. Thomas A. Hicks and Dr. James R. Hicks have moved their offices from Franklin St. to 55 Allen St., Buffalo.

---

Dr. F. E. Fronczak, Health Commissioner of Buffalo, has returned from the American Congress of Hygiene and Demography at Washington.

Dr. Max Breuer of Buffalo, has returned from Europe.

Dr. Geo. A. Himmelsbach of Buffalo, has returned from Europe.

Dr. Fred J. Parmenter of Buffalo, spent two weeks in the Canadian forests during September.

Dr. W. Scott Renner of Buffalo, has returned from Europe.

Dr. Lawrence Hendee of Buffalo, spent September on Fourth Lake.

Dr. Carroll L. Roberts of Buffalo, has returned from a fifteen-months' study tour of Europe.

Drs. James A. Gardner and James B. Cross of Buffalo, have opened their new office in suite 504, General Electric Building.

Dr. W. H. Woodbury of Buffalo, has returned from Europe.

Dr. Sherman Voorhees of Elmira spent the last half of August in Boston and Bar Harbor.

Dr. Charles F. Abbott of Elmira has returned from a trip to New York and Philadelphia.

Dr. J. A. Lichty of Pittsburg, called on the editor Sept. 11.

Mr J. C. Bradley has retired from the position of Manager of the Denver Chemical Manufacturing Co. He is succeeded by Mr. Harold B. Scott, a Yale graduate who has had a wide varied and successful business experience. We wish to express our aperciation of Mr. Bradley's many courtesies and to extend to him our best wishes. And, to Mr. Scott, we extend our greeting and congratulations—as also to the firm.

H. R. Carson, Sc. M., Ph. G., M.D., of Northwestern University, will assist Dr. J. E. Walker at the Steuben Sanitarium, Hornell, N. Y. Dr. Carson was an interne at St. Luke's Hospital, Chicago, and was engaged in practice in the same city. He comes into our territory highly recommended.

Dr. James S. Porter of Buffalo, has returned from a fishing trip at Burlidge Falls, Ont.

Dr. George W. Batt, formerly of 441 E. Genesee St., Buffalo, has taken the office of Dr. Roy J. Juhre at Hinsdale. Dr. Juhre has returned to Buffalo, where he expects to open an office soon.

